# Problematic Social Networking Site Use and Comorbid Psychiatric Disorders: A Systematic Review of Recent Large-Scale Studies

**DOI:** 10.3389/fpsyt.2018.00686

**Published:** 2018-12-14

**Authors:** Zaheer Hussain, Mark D. Griffiths

**Affiliations:** ^1^School of Human Sciences, College of Life and Natural Sciences, University of Derby, Derby, United Kingdom; ^2^International Gaming Research Unit, Psychology Department, Nottingham Trent University, Nottingham, United Kingdom

**Keywords:** attention deficit and hyperactivity disorder, obsessive compulsive disorder, depression, anxiety, problematic social media use, social media addiction

## Abstract

**Background and Aims:** Research has shown a potential association between problematic social networking site (SNS) use and psychiatric disorders. The primary objective of this systematic review was to identify and evaluate studies examining the association between problematic SNS use and comorbid psychiatric disorders.

**Sampling and Methods:** A literature search was conducted using the following databases: PsychInfo, PsycArticles, Medline, Web of Science, and Google Scholar. Problematic SNS use (PSNSU) and its synonyms were included in the search. Information was extracted based on problematic SNS use and psychiatric disorders, including attention deficit and hyperactivity disorder (ADHD), obsessive compulsive disorder (OCD), depression, anxiety, and stress. The inclusion criteria for papers to be reviewed were (i) being published since 2014 onwards, (ii) being published in English, (iii) having population-based studies with sample sizes >500 participants, (iv) having specific criteria for problematic SNS use (typically validated psychometric scales), and (v) containing empirical primary data reporting on the correlation between PSNSU and psychiatric variables. A total of nine studies met the predetermined inclusion and exclusion criteria.

**Results:** The findings of the systematic review demonstrated that most research has been conducted in Europe and all comprised cross-sectional survey designs. In eight (of the nine) studies, problematic SNS use was correlated with psychiatric disorder symptoms. Of the nine studies (some of which examined more than one psychiatric symptom), there was a positive association between PSNSU and depression (seven studies), anxiety (six studies), stress (two studies), ADHD (one study), and OCD (one study).

**Conclusions:** Overall, the studies reviewed showed associations between PSNSU and psychiatric disorder symptoms, particularly in adolescents. Most associations were found between PSNSU, depression, and anxiety.

## Introduction

Global use of social networking sites (SNSs) is growing with over 2 billion users worldwide. North America and Europe rank highest in terms of SNS usage with 70% and 66% social media penetration rates, respectively (1). Research has shown that amongst American teenagers, *YouTube, Instagram*, and *Snapchat* are the most popular SNSs, and 45% of teenagers stated that they are online almost constantly (2). Given the broad spectrum of SNS use, it is important to understand the potential risks involved in problematic SNS use. Public health concerns have been voiced by the World Health Organization (3) regarding the propensity of problematic SNS behaviors associated with addiction among a minority of users. Empirical studies have shown that problematic internet users spend more time online than non-problematic users (4, 5). Problematic SNS use (PSNSU) has been conceptually defined as a disorder that does not involve ingestion of a psychoactive substance and shares qualities related to a behavioral addiction (6). Other researchers (7) have used the term SNS addiction. Andreassen and Pallesen (7) define SNS addiction as being overly concerned about SNSs, driven by a strong motivation to log on to or use SNSs, and to devote so much time and effort to SNSs that it impairs other social activities, education and/or occupation, interpersonal relationships, and/or psychological health and well-being. Behavioral addictions, as defined by Griffiths (6, 8), are behaviors that comprise six components: salience, mood modification, tolerance, withdrawal symptoms, conflict, and relapse. These components have been reported among SNS users in several studies [e.g., (9–11)]. The prevalence of PSNSU varies among populations ranging from 1.6% in Nigeria (12), 4.5% in Hungary (13), 8.6% in Peru (14), and 12% in China (15), although only the study by Banyai et al. (13) used a nationally representative sample. These findings show that PSNSU is an increasing concern across cultures.

SNSs allow users to communicate with individuals around the world, to keep in touch with family and friends providing a feeling of connection (16) and increased online social well-being (17). Research has demonstrated that connectivity on SNSs can facilitate well-being offline (18). However, increased monitoring, compulsive checking, and excessive engagement with these websites may be leading to the emergence of psychiatric disorder symptoms among a minority of individuals (19). Maladaptive SNS use has been identified as a potential mental health problem (20) with such claims being supported by empirical findings showing that PSNSU is associated with health-related, interpersonal, and educational problems (7, 21). Empirical research has also shown PSNSU to be negatively associated with life satisfaction (22, 23) and low quality of life (24). Recent research (25, 26) has shown that narcissistic individuals spend more time on SNSs, and those individuals who possess high levels of Dark Triad personality traits may employ SNSs to execute “cheater strategies” that help them achieve their interpersonal and social goals despite their anti-social personality traits (26, 27). Halpern et al. (28) reported that narcissistic individuals take “selfies” (self-portraits uploaded and shared on social media) more frequently over time which leads to increased levels of narcissism. In a comprehensive study examining worldwide selfie-related accidental mortality, Jain and Mavani (29) reported that between 2014 to mid-2016, 75 individuals died while attempting a selfie in 52 incidents worldwide, with the mean age of the victims being 23.3 years and 82% were male. Research by Nesi and Prinstein (30) examined longitudinal associations between adolescents' digital status seeking (an investment of significant effort into the accumulation of online indicators of peer status and approval) and health-risk behaviors. The results showed that digital status seekers engaged in higher levels of substance use and sexual risk behavior 1 year later. Sarabia and Estévez (31) examined sexualized behaviors displayed by Spanish adolescents on their *Facebook* profiles, and results showed that girls tried to please others via seduction to obtain social approval while boys presented themselves as seducers. Taken as a whole, research is beginning to show how SNSs may influence behavior which is concerning given the wide usage and popularity of these websites.

Due to the widespread use of SNSs, research concerning its implications for mental health necessitates scientific review. There may be severe mental health implications for the minority of individuals experiencing PSNSU. Previous systematic reviews have focused on pathological internet use and psychopathology (32), problematic internet use (33, 34), and problematic smartphone use and anxiety and depression symptoms (35). However, there are no recent review papers overviewing PSNSU and psychiatric symptoms. A focused systematic review examining the associations between PSNSU and psychiatric disorder symptoms is therefore warranted and is necessary for prevention and treatment work. It is important to examine and highlight the symptoms of psychiatric disorder before they become fully developed disorders. The main aims of this focused review were to (i) review the scientific literature concerning SNSs and psychiatric disorders, (ii) identify and evaluate studies performed on the correlation between PSNSU and comorbid psychiatric disorders, (iii) to map the geographical distribution of studies, and (iv) assess the quality of the studies. Based on previous literature, the following psychiatric disorders were included: attention deficit and hyperactivity disorder (ADHD), obsessive compulsive disorder (OCD), depression, anxiety, and stress.

## Methods

**Figure 1:**
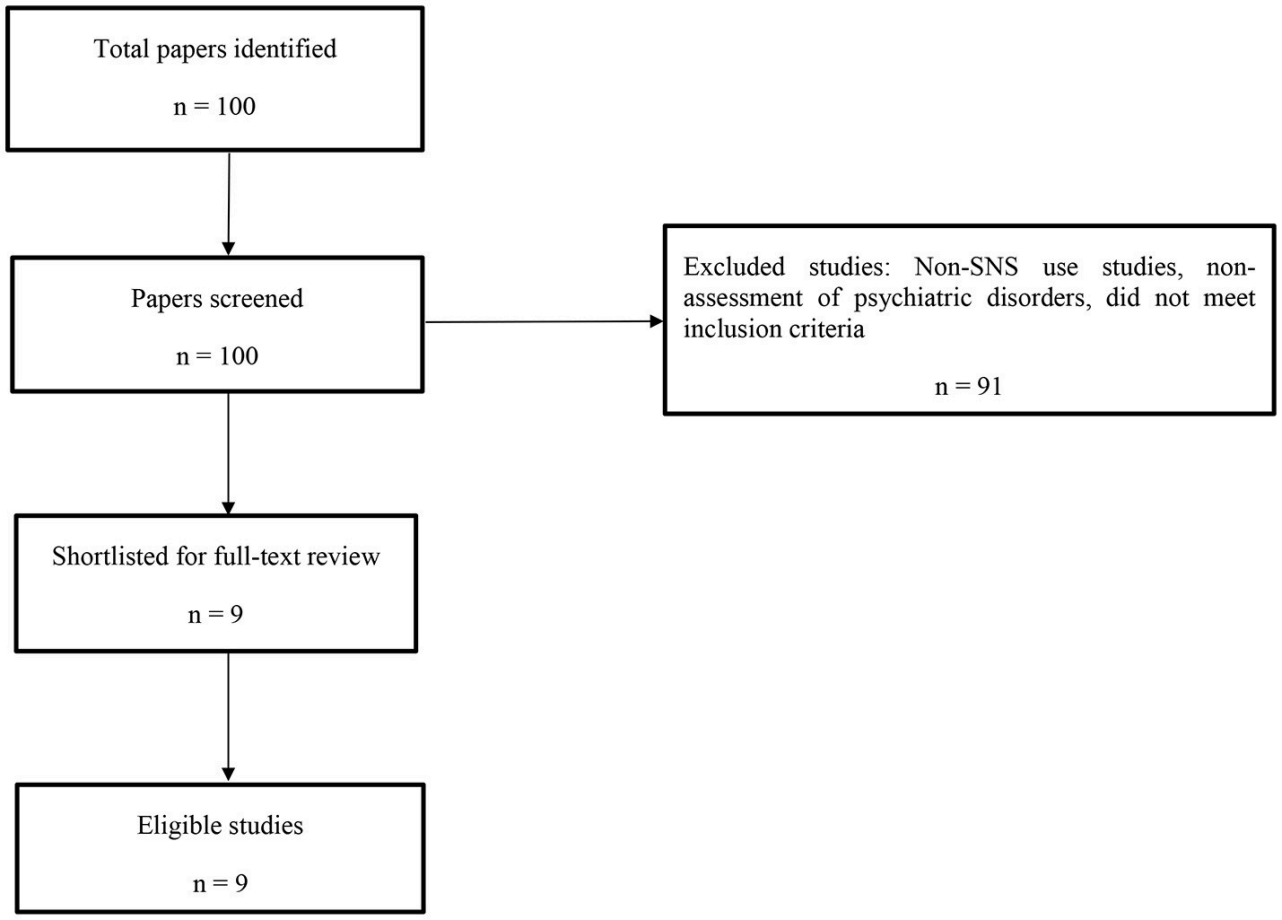
Flow diagram of review process.

The preferred reporting items for systematic reviews and met-analysis [PRISMA; (36)] were closely adhered to during the review process (see Figure 1). A comprehensive literature search was conducted using PsychInfo, PsycArticles, Medline, Web of Science, and Google Scholar databases. Reference lists of retrieved articles and review papers were also examined for any further studies. The main keywords used in the search were “problematic social networking site,” “problematic social media use” or “social networking site addiction,” “social media addiction,” “social networking site dependence,” “social media dependence,” “pathological social networking site use,” “pathological social media use,” “compulsive social networking site use,” “compulsive social media use,” “excessive social networking site use” and “excessive social media use” combined with the keywords of “attention deficit and hyperactivity disorder/ADHD” or “obsessive compulsive disorder/OCD,” “depression” or “anxiety” or “stress.”

Studies were systematically and independently reviewed by the authors and assessed regarding the study type, study population, methodology, measures used, and interpretation of results. The inclusion criteria for papers to be reviewed were (i) being published since 2014 onwards, (ii) being published in English (the native language of the present authors), (iii) having population-based studies with sample sizes >500 participants, (iv) having specific criteria for PSNSU (typically validated psychometric scales), and (v) containing empirical primary data reporting on the correlation between PSNSU and psychiatric variables. Studies were excluded if they comprised case studies and/or comprised treatment assessment. Papers were screened based on the titles and abstracts reporting on the topics of interest. Thereafter, papers were selected based on scientific relevance of the study and included following full text assessment. Studies were evaluated if there was an observation of a full association and significance level. Full association was considered when a correlation was found for PSNSU and specific psychiatric disorder symptoms following bivariate or multivariate analysis. The geographical distribution of studies was also mapped.

After deleting duplicate studies, a total of 100 papers were screened and identified via the systematic search strategy employed. As a result, 91 papers were excluded because they (i) were based on other internet-related topics and not social media use, (ii) did not assess psychiatric disorder symptoms, and (iii) did not meet the inclusion criteria (see Figure 1 for a flow diagram of the review process). Thus, there were nine papers included in the present review. The characteristics of the studies included in the review (see Table 1) are discussed below. Some studies are referred to in more than one section due to assessing more than one psychiatric disorder symptom. The results section also briefly reports the key findings of a further nine studies that met all the inclusion criteria apart from sample size.

**Table 1:** Study details and results.

**Authors (year and country of study)**	**Sample size (age range)**	**Study variables**	**Scale used to assess P-SNS-U**	**Results with PSNSU**	**Effect sizes: Bivariate results**	**Effect sizes: multivariate results**
Andreassen et al. [(37), Norway]	23,533 (16–88 years)	PSNSU, ADHD, OCD, anxiety, depression	Bergen social media addiction scale Andreassen et al. (38)	Positive and significant association with all study variables	ADHD *r* = 0.41 OCD *r* = 0.33 Anxiety *r* = 0.34 Depression *r* = 0.19	ADHD β = 0.268 OCD β = 0.147 Anxiety β = 0.074 Depression β = −0.018
Banyai et al. [(13), Hungary]	5,961 (15–22 years)	PSNSU, depression, self-esteem, weekly SNS use	Bergen social media addiction scale Andreassen et al. (38)	Positive association between with depression	n/a	n/a
Kircaburun et al. [(39), Turkey]	Study 1: 804 (14–21 years), Study 2: 760 (18–40 years)	PSNSU, cyberbullying, depression, happiness, self-esteem	Social media use questionnaire (40)	Positive and significant association with depression	Study 1: Depression *r* = 0.37, Study 2: Depression *r* = 0.22	n/a
Pontes [(41), Portugal]	509 (10–18 years)	SNS addiction, depression, anxiety, stress,	Bergen facebook addiction scale Andreassen et al. (38)	Positive and significant association with depression, anxiety, and stress	Depression *r* = 0.33, Anxiety *r =* 0.31, Stress *r* = 0.36	SNS addiction contributed toward the severity of: Depression (β = 0.27) Anxiety (β = 0.25) Stress (β = 0.26)
Shensa et al. [(42), USA]	1,749 (19–32)	PSNSU, depression	Bergen facebook addiction scale (38)	Positive and significant association with depression	n/a	Significantly associated with a 9% increase in odds of depressive symptoms: AOR = 1.09; 95% CI: 1.05, 1.13; *p* < 0.001
Van Rooij et al. [(43), The Netherlands]	3,945 (12–15 years)	PSNSU, depression, loneliness, anxiety, self-esteem, life satisfaction	The Compulsive Internet Use Scale (CIUS) (44)	Positive and significant association with depression and anxiety	Depression *r* = 0.45 Anxiety: new situation *r* = 0.22, Anxiety: general *r* = 0.17	Depression β = 0.32
Worsley et al. [(45), UK]	1,029 (17–25 years)	PSNSU, anxiety, depression	Bergen social media addiction scale (38)	Positive and significant association with depression and anxiety	Depression *r* = 0.27 Anxiety *r* = 0.15	Depression B = 0.17 Anxiety B = 0.05
Atroszko et al. [(46), Poland]	1,157 (mean age 20 years: age range unreported)	Facebook addiction, personality traits, self-esteem, self-efficacy, narcissism	Bergen facebook addiction scale (38)	Positive and significant association with anxiety and stress	Anxiety *r* = 0.19, Stress *r* = 0.22	Anxiety β = 0.16
Dhir et al. [(47), India]	Study A: 1,554, (12–18 years), Study B: 1,144 (12–18 years)	Compulsive SNS use, SNS fatigue, anxiety, depression, fear of missing out	Bergen facebook addiction scale (38)	Indirect positive association (via SNS fatigue with depression and anxiety)	n/a	Study A: Depression β = 0.25 Anxiety β = 0.23 Study B: Depression β = 0.22 Anxiety β = 0.12

*Effect sizes are factor results reported with PSNSU unless otherwise stated; β, standardized regression coefficient; B, Unstandardized regression coefficient; AOR, Adjusted odds ratio; CI, Confidence interval*.

## Results

### Description of Included Studies and Geographical Distribution

All nine of the studies were cross-sectional survey studies. Most studies (*n* = 7) targeted adolescent and/or emerging adult groups (13, 39, 41, 43, 45–47), while two studies had a mainly adult sample (37, 42). Only two studies used a nationally representative population [i.e., (13, 42)]. All studies examined both genders and the sample sizes ranged from 509 to 23,533. The majority of studies (*n* = 7) were carried out in European countries (Hungary, Netherlands, Norway, Poland, Portugal, Turkey, United Kingdom), one study was carried out in the United States [i.e., (42)], and one in India [i.e., (47)]. Table 1 summarizes further information about the included studies.

### Methods of Assessing Problematic Social Networking Site Use

Most studies (*n* = 7) assessed PSNSU using the Bergen Social Media Addiction Scale [BSMAS; (37)] or its predecessor, the Bergen Facebook Addiction Scale [BFAS; (38)]. The BSMAS contains six items reflecting the core components of addiction (6). The six BSMAS questions were modified by replacing the word “*Facebook”* in the original BFAS with the words “social media.” Participants rate all items on a 5-point Likert scale (where 1 = very rarely, 2 = rarely, 3 = sometimes, 4 = often, 5 = very often). Example adapted questions include: “How often during the last year have you felt an urge to use social media more and more?” and “How often during the last year have you used social media in order to forget about personal problems?” Various studies have reported that the BFAS and adapted BSMAS have good psychometric properties [e.g., (48–51)]. The two remaining studies used the Social Media Use Questionnaire (40) and the Compulsive Internet Use Scale [CIUS; (44)], the latter of which does not specifically assess problematic social media use. Table 1 provides details of measurement instruments used by studies to assess PSNSU.

### Problematic Social Networking Site Use and ADHD

Only one study [i.e., (37)] examined the associations between PSNSU and ADHD. The authors assessed ADHD using the Adult ADHD Self-Report Scale [ASRS-Version 1.1; (52)]. Significant and positive associations between PSNSU and ADHD were reported. The bivariate correlation was *r* = 0.41, and the multivariate association was β = 0.268.

### Problematic Social Networking Site Use and OCD

Only one study (37) examined the associations between PSNSU and OCD. The authors assessed OCD using the Obsessive-Compulsive Inventory-Revised [OCI-R; (53)]. The results showed a significant and positive association between PSNSU and OCD. The bivariate correlation was *r* = 0.33, and the significant multivariate association was β = 0.147.

### Problematic Social Networking Site Use and Depression

Seven studies examined the associations between PSNSU and depression [i.e., (37, 39, 41–43, 45, 47)]. A significant and positive association between PSNSU and depression was reported in six studies [i.e., (37, 39, 41–43, 45)]. One study [i.e., (47)] reported an indirect positive association between PSNSU and depression. Bivariate correlations were typically in the range of 0.19–0.45. Multivariate associations showed betas ranging from −0.018 to 0.32. Each of these studies finding a positive association between PSNSU and depression used different scales to assess depression: (i) Andreassen et al. (37) used the depression scale of the Hospital Anxiety and Depression Scale (HADS; 53); (ii) Dhir et al. (47) used the Depression Scale [DEPS; (54)]; (iii) Kircaburun et al. (55) used the depression sub-scale of the Short Depression-Happiness Scale [SDHS; (56)]; (iv) Pontes (41) used the depression scale of the Depression Anxiety and Stress Scales−21 [DASS-21; (57)]; (v) Shensa et al. (42) used the Patient-Reported Outcomes Measurement Information System depression scale [PROMIS; (58)]; (vi) Van Rooij et al. (43) used the six-item Depressive Mood List (59, 60); and (vii) Worsley et al. (45) used the Patient Health Questionnaire [PHQ-9; (61)]. One study (13) reported high levels of depression among participants who displayed PSNSU but no bivariate or multivariate associations were reported in the results. Bányai et al. (13) used the Center of Epidemiological Studies Depression-Scale [CES-D; (62)].

### Problematic Social Networking Site Use and Anxiety

Six studies [i.e., (37, 41, 43, 45–47)] examined the associations between PSNSU and anxiety. A significant and positive association between PSNSU and anxiety was reported in five studies [i.e., (37, 41, 43, 45, 46)]. One study [i.e., (47)] reported an indirect positive association between PSNSU and anxiety. When examining the bivariate correlations, they were typically in the range of 0.15–0.34. Multivariate associations showed betas that ranged from 0.05 to 0.074. The studies assessed anxiety with different scales: (i) Andreassen et al. (37) used the anxiety scale of the Hospital Anxiety and Depression Scale [HADS; (63)]; (ii) Atroszko et al. (46) used the shortened Polish version of Liebowitz Social Anxiety Scale [LSAS; (64, 65)]; (iii) Dhir et al. (47) used the Social Anxiety Scale for Adolescents [SAS-A; (66)]; (iv) Pontes (41) used the anxiety scale of the DASS-21 (57); (v) Van Rooij et al. (43) used the social anxiety scale (67); and (vi) Worsley et al. (45) used the anxiety dimension of the Relationship Questionnaire [RQ; (68)].

### Problematic Social Networking Site Use and Stress

Two studies [i.e., (41, 46)] examined the associations between PSNSU and stress with a significant and positive association between PSNSU and stress being reported in both of them. The bivariate correlations ranged from 0.22 to 0.36, and multivariate associations showed betas ranging from 0.16 to 0.26. Atroszko et al. (46) assessed stress using the Perceived Stress Scale [PSS-4; (69)], and Pontes (41) assessed stress by using the stress scale of the DASS-21 (57).

### Problematic Social Networking Site Use in Other Studies (Sample Sizes < 500 Participants)

During the initial literature search, nine studies were found that met all the inclusion criteria apart from having sample sizes above 500 participants. Most of these studies' findings concurred with those outlined in the previous sections. Two studies {i.e., (70) [*n* = 289]; (71) [*n* = 283]} examined the associations between PSNSU and ADHD. Significant and positive associations between PSNSU and ADHD were reported in both studies. Seven studies {e.g., (72) [*n* = 253]; (73) [*n* = 442]; (74) [*n* = 374]; (55) [*n* = 344]; (75) [*n* = 197]; (76) [*n* = 365]; (77) [*n* = 334]} examined the associations between PSNSU and depression with significant and positive associations being reported in all seven studies. Five studies {(78) [*n* = 207]; (79) [*n* = 451]; (80) [*n* = 243]; (75) [*n* = 197]; (77) [*n* = 334]} examined the associations between PSNSU and anxiety with a significant and positive association being reported in all the studies. Two studies {(81) [*n* = 499]; (75) [*n* = 197]} examined the associations between PSNSU and stress with a significant and positive association being reported in both of them. Finally, one study {i.e., (82) [*n* = 215]} reported a negative association between PSNSU and depression.

## Discussion

The present systematic review examined the associations between PSNSU and psychiatric disorder symptoms in nine studies that met the inclusion criteria. Of the nine studies, there was a positive association between PSNSU and depression (seven studies), anxiety (six studies), stress (two studies), ADHD (one study), and OCD (one study). The general findings of the review suggest that PSNSU co-occurs with all psychiatric disorders that have been examined (i.e., ADHD, OCD, depression, anxiety, and stress). Similar results were also found in the nine studies that met all the criteria for inclusion apart from having samples < 500 participants. The review showed that depression was significantly associated with PSNSU in seven of the reviewed studies. Based on the effect size conventions (83), these relationships generally had small to medium bivariate effect sizes across the studies. The review also showed that anxiety was significantly associated with PSNSU in six of the studies, and that the relationships generally had small bivariate effect sizes across the studies. It is important to note that only a small number of studies have examined the relationship between PSNSU and symptoms of psychiatric disorder. The association between the variables will need to be re-assessed in the near future once there are further empirical studies. Based on the studies reviewed, it is plausible to suggest that participants with PSNSU are at higher risk of psychiatric disorders. However, it is also possible that those with psychiatric illness symptoms may be more prone to PSNSU. Different measurement instruments were used to assess PSNSU with the BSMAS (and its predecessor, the BFAS) being the most utilized psychometrically validated measure. The review also showed that adolescent samples were the most utilized group with only two studies targeting the general population. This shows that future research needs to examine SNS use in the general adult population.

### Explanations for Psychiatric Disorder Symptoms Relations With PSNSU

The reviewed studies provided some explanations for the relationship between PSNSU and psychiatric disorder symptoms. Andreassen et al. (37) suggested individuals over-using SNSs may experience a constant urge to check their social networks for new information and updates because of the fear of missing out (84–87). Andreassen et al. (33) also suggest that those with ADHD symptoms may use SNSs excessively due to the constant updates from these platforms and beeping and vibrating smartphones [SNSs are now typically accessed via smartphones; (88)]. Shensa et al. (42) suggest that individuals with greater PSNSU levels neglect other constructive aspects of their lives which could contribute to depression symptoms. Also, it may be that individuals experiencing depressive symptoms are more prone to PSNSU. It is important to note that Shensa et al. (42) found that social media use frequency (but not social media time) was significantly and independently associated with depressive symptoms. This suggests that it may be how individuals use social media (and not how much) that is associated with depressive symptoms. Atroszko et al. (46) concluded that *Facebook* dependent individuals crave social interactions and self-validation, and that compulsive *Facebook* activity may generate stress. Worsley et al. (45) inferred that young users may be excessively using SNSs in order to avoid or reduce depression and anxiety. This provides support for the work of Seabrook et al. (89) who claimed that there is a possibility that the relationship between PSNSU and depression symptoms may be bi-directional. Taken together, these findings provide interesting insights into the mechanisms of SNS use and the influence they have on the individual user (especially those who are susceptible to psychiatric disorders). Some of the studies suggest that the experiences of SNS users and how they respond to the rewarding aspects of SNS use may be a defining factor in the occurrence of PSNSU. Furthermore, the studies pave the way for future research to investigate specific cognitive factors (e.g., fear of missing out) and motivational factors (e.g., escapism) associated with PSNSU. Brand et al. (90) have argued that future studies should explicitly address the personality profiles among different forms of Internet-related disorders because this may reveal common and unique correlates of dysfunctional use of SNSs.

External factors (such as work/home environments) that may affect the relationship between SNS use and psychiatric disorder symptoms should be taken into consideration. SNSs can serve as a platform with positive and/or negative effects on users' psychological health and well-being. Given that adolescents are using SNSs at a high rate worldwide, as a specific group they are more likely to experience co-morbid psychiatric symptoms (42). Research evidence suggests that increased levels of internet use can cause the types of psychiatric symptoms included in the present review among a minority of individuals (34). For instance, problematic technology use (internet use, social media use, computer use, mobile phone use, and smartphone use) have all been associated with greater subsequent levels of depression, stress, anxiety and sleep disturbance among a minority of individuals (34, 91–94). Future research should focus on prevention programs and interventions to ameliorate the potential adverse consequences among this minority of problematic SNS users. The findings from this review benefits researchers and clinicians in helping pinpoint symptoms of psychiatric disorders during intervention programs and consultations.

## Limitations

The limitations of the studies covered in the review is that all of them were cross-sectional surveys. Although this type of study design is arguably reliable in determining such associations (despite well-known biases such as memory recall and social desirability), it is not possible to ascertain causality. Longitudinal study data is required to assess causal interactions between variables examined in the present review. Most of the studies were based on European samples (and even then, only data from seven countries was collected) which limits the generalizability of the findings. Consequently, the results cannot be interpreted in the context of other cultures and continents. Although the present review only included large scale studies, only two studies had very large sample sizes over 5,000 participants [i.e., (13, 37)], and only two studies collected data from representative populations [i.e., (13, 42)]. Further research is therefore needed using much larger and representative samples. Overall, the studies reviewed here showed associations between PSNSU and psychiatric disorder symptoms, particularly in adolescents. Most associations were found between PSNSU, depression, and anxiety. Future research should focus on other psychiatric disorder symptoms using different study methods. Future studies should also focus on the potential negative consequences of SNS use and psychiatric disorders by implementing methodologies other than cross-sectional surveys. An interesting angle for future research would be to assess associations between different behavioral addictions because there may be common etiologies and psychopathologies.

## Conclusions

This systematic review demonstrates that PSNSU and psychiatric disorder symptoms co-occur, particularly in adolescents. Most associations were found between PSNSU, depression, and anxiety. Future research using different methods (other than cross-sectional surveys) and representative samples is required.

## Author Contributions

ZH and MG: Paper concept and design; writing and finalization of the manuscript; approved the final manuscript. ZH: Literature searches, initial analyses of studies, and initial drafting of the manuscript.

### Conflict of Interest Statement

The authors declare that the research was conducted in the absence of any commercial or financial relationships that could be construed as a potential conflict of interest.
